# A Novel Bio-Inspired Bat Node Scheduling Algorithm for Dependable Safety-Critical Wireless Sensor Network Systems

**DOI:** 10.3390/s24061928

**Published:** 2024-03-17

**Authors:** Issam Al-Nader, Aboubaker Lasebae, Rand Raheem, Gerard Ekembe Ngondi

**Affiliations:** 1 Faculty of Science & Technology, Department of Computer Science, Middlesex University, The Burroughs, London NW4 4BT, UK; a.lasebae@mdx.ac.uk (A.L.); r.h.raheem@mdx.ac.uk (R.R.); 2 Computer Science Department, School of Statistics and Computer Science, Trinity College, Dublin D02 PN40, Ireland; gerard.ekembe@tcd.ie

**Keywords:** IoT sensor system, WSN, smart sensing for safety, dependable WSN, scheduling algorithms, real-time systems, QoS in WSNs

## Abstract

The multi-objective optimization (MOO) problem in wireless sensor networks (WSNs) is concerned with optimizing the operation of the WSN across three dimensions: coverage, connectivity, and lifetime. Most works in the literature address only one or two dimensions of this problem at a time, except for the randomized coverage-based scheduling (RCS) algorithm and the clique-based scheduling algorithm. More recently, a Hidden Markov Model (HMM)-based algorithm was proposed that improves on the latter two; however, the question remains open if further improvement is possible as previous algorithms explore solutions in terms of local minima and local maxima, not in terms of the full search space globally. Therefore, the main contribution of this paper is to propose a new scheduling algorithm based on bio-inspired computation (the bat algorithm) to address this limitation. First, the algorithm defines a fitness and objective function over a search space, which returns all possible sleep and wake-up schedules for each node in the WSN. This yields a (scheduling) solution space that is then organized by the Pareto sorting algorithm, whose output coordinates are the distance of each node to the base station and the residual energy of the node. We evaluated our results by comparing the bat and HMM node scheduling algorithms implemented in MATLAB. Our results show that network lifetime has improved by 30%, coverage by 40%, and connectivity by 26.7%. In principle, the obtained solution will be the best scheduling that guarantees the best network lifetime performance as well as the best coverage and connectedness for ensuring the dependability of safety-critical WSNs.

## 1. Introduction

Wireless technological advances enabled a new networking system known as the Internet of Things (IoT). Such systems allow data to be exchanged among other devices, sensors, and software through the Internet [1,2]. A wireless sensor network (WSN) is a distributed system composed of sensor nodes and one or more powerful nodes called base stations [3]. The main objective of a WSN is to detect events of interest and then report those detected events through a chain of connected sensor nodes to the base station [4]. Such types of networks have inherently limited resources, e.g., limited memory, processing, and energy [5,6,7]. Due to their easy deployment, they are used in a wide variety of applications, specifically in the field of safety-critical systems [8,9]. For example, a WSN can be deployed on the top of a volcano where human intervention is impossible or to monitor the radiation leakage at a nuclear plant station, which poses a risk to human lives [10]. Hence, when a WSN is designed for safety-critical applications, another level of complexity is added as rigid time/deadline requirements are to be respected [11,12]. Such resources need to be optimized for WSNs to function efficiently. Enhancing the performance of multiple resources in the same system is considered a multi-objective optimization problem or an NP-hard problem for WSNs [13].

The objective of optimizing the performance of safety-critical WSNs is to improve their dependability, which is determined by three requirements: coverage, connectivity, and lifetime. In this paper, we will say that “A WSN is dependable for a given application if its maximum lifetime is equal to or greater than its expected service time”. Several works in the literature attempt to optimize WSNs by focusing on only one or two performance requirements. Below, we discuss only works that look at all three requirements together. The randomized coverage-based scheduling (RCS) algorithm splits the network into different sub-networks, where nodes in the WSN join each of these sub-networks randomly. The sub-networks alternate between ON and OFF states. The problem with this is that RCS forces nodes to be turned ON, although a node could be scheduled to be in an OFF state. This exacerbates the WSN by creating hot spot nodes that will consume energy faster and die prematurely, leading to the partition of the network whereby events can be detected but not reported back to the base station [13].

The Hidden Markov Model (HMM)-based node scheduling algorithm [14] addresses the limitation introduced by the RCS algorithm [13] by balancing the workload of the node duty cycle. The HMM node scheduling algorithm schedules the receive and send states of nodes such that the node that has the highest energy consumption will be scheduled for receiving at a certain round. Although this solution has improved network lifetime while maintaining network coverage and connectivity through RCS, it comes with its challenges. Notably, it is limited to local minima and local maxima, whereby the obtained solution is limited to the information made available by the sensor nodes’ ON states (send, receive, and idle). We hypothesize that if more information were available, a better schedule could be devised. Therefore, the motivation is to provide a new scheduling solution coupled with a Pareto sorting algorithm that shall address the previous limitations, e.g., considering the three objectives altogether (connectivity, coverage, and lifetime). The importance of this work comes from the need to have functional yet dependable safety-critical systems in WSNs [8,9,10].

In this paper, the contribution, for the first time, is a novel bat node scheduling algorithm to optimize the node scheduling of WSNs along the three requirements of coverage, connectivity, and lifetime. This notably permits the exploration of a larger search space to find the best scheduling solution. Furthermore, the solution space is then organized by the Pareto sorting algorithm, whose output coordinates are the distance of each node to the base station and the residual energy of the node. Our bat node scheduling algorithm works on a continuous search space using a probability perception mathematical model. We compare the performance of our bat node scheduling algorithm against the HMM node scheduling algorithm [14] using MATLAB. The results show significant improvements in all three requirements. This method can provide a better solution faster than other natural heuristic algorithms; however, it requires more computation in most network environments.

This paper is structured as follows: Section 2 discusses related work represented in the literature. Section 3 introduces the proposed bat with its mathematical representation, with Section 3.1 presenting the formulation of the problem and Section 3.2 discussing the use-case scenario of the bat node scheduling algorithm. Section 4 covers the experimentation and the discussion of the results of this work. Finally, Section 5 presents our conclusion.

## 2. Related Work

To the authors’ best knowledge, no work in the literature has investigated the use of the bio-inspired method for node scheduling algorithms [15]. There are, however, several bio-inspired computation approaches for optimizing only energy, such as the genetic algorithm [16]. In addition, work such as [17] uses swarm intelligence (SI) for the multi-objective optimization (MOO) problem. The basic idea of the SI model is that working collectively and collaboratively is much more effective than the work of individual members of that SI in achieving a common goal. For example, fish schooling and bee and ant colonies solve the MOO problem. Most works based on bat algorithms in WSNs focus on node localization and work at the network/routing layer; they are not used at the application/management layer where node scheduling algorithms are found. The study [18] contains a comprehensive survey of energy management and node scheduling schemes, wherein only one paper is mentioned that uses a bat algorithm to improve network coverage [19].

The study [19] proposes a bat algorithm called Target-Connected Coverage (TCC), which aims to ensure that every wireless sensor node is connected to the sink node through at least one communication path at all times. This is performed using a game-theoretic approach. The TCC algorithm [19] works by proposing a couple of bats that are based on a binary search space. The first bat’s main goal is to find the active node in the WSN, and the second bat’s goal is to use the active node to find at least one path to the sink node to report data while maintaining connectivity. A limitation of [19] is that it does not consider any node scheduling scheme; hence, nodes were assumed to always be in an active state (ON). In addition, the authors in [19] considered both connectivity and network lifetime requirements but not coverage.

The survey [18] presents a taxonomy of QoS-aware energy management for WSNs, focusing on node scheduling techniques. The authors in [18] present their classification to evaluate various node scheduling techniques based on very important requirements such as coverage, connectivity, fault tolerance, and WSN security. In addition, ref. [18] stated design issues and challenges that surround the implementation of the node scheduling approach, which, if mitigated, will help achieve WSN QoS requirements in several applications. In particular, they presented a classification of node scheduling schemes based on coverage requirements, under which falls our proposed bat-based scheduling algorithm. Intuitively, if network coverage is taken into consideration, this would guarantee better network connectivity. Furthermore, ref. [18] proposes an energy management life-cycle model along with an energy conservation pyramid to extend the network lifetime in WSNs. The pyramid model is composed of three key energy conservation techniques, which are: duty cycle, data aggregation, and mobility. In general, node scheduling utilizes the duty cycle of sensor nodes (sleep/awake) to conserve energy, thereby extending the lifetime of the network. The energy pyramid module classifies the latest scheduling methods in the field based on their objectives and minimization parameters.

Other bio-inspired flocking techniques were used to detect anomalies in the stream of data [20] in WSNs. In this paper [20], the authors solve the problem of topology management in WSNs using the flocking model-based bio-inspired algorithm. First, the solution is restricted to 2D space. Second, the authors made use of the density distribution of the sensor nodes in the WSN. The flocking model is used in which the agent is moving into a space in a fixed time. Hence, when two agents of similar density encounter each other, they form a flock. Since these flocks form a swam/group, offline computation can easily be avoided using this approach. The approach assumes the agents are on when sensor nodes are active, which is not the case in WSNs as sensor nodes alternate between ON and OFF, rendering the density changeable.

Having delved into the latest state-of-the-art node scheduling literature, it can be concluded that most scheduling algorithms focus on considering only two requirements and do not address all three requirements of the WSN altogether, i.e., connectivity, coverage, and network lifetime. Most works address each requirement in isolation, e.g., [4,11,12,18,21,22,23,24,25,26,27,28] do not consider network coverage; other works such as [6,29] consider network coverage but without ensuring a good level of network connectivity; and [6] uses a very simple WSN scenario that lacks tests on realistic and complex WSNs. To the authors’ best knowledge, there are two published works that consider addressing the three objectives collectively: the RCS algorithm [13] and clique-based node scheduling [13,30] have shown that partitions can occur further away from the base station as the deaths of subsequent nodes can result in live nodes attempting to compensate for the drop-in coverage and connectivity in the system (cf. Section 3). The clique-based approach [30] relies on a complex node localization and clustering mechanism. While [30] guarantees maximum connectivity and coverage ratio, this comes at the expense of heavy computation, thus making the verification of the algorithm more difficult. Given the limitations in the state-of-the-art literature, our contribution is to utilize a bio-inspired computation model to provide a bat-based scheduling solution that obtains a better solution from a new representation of the search space.

## 3. The Bat Node Scheduling Algorithm

**Figure 1:**
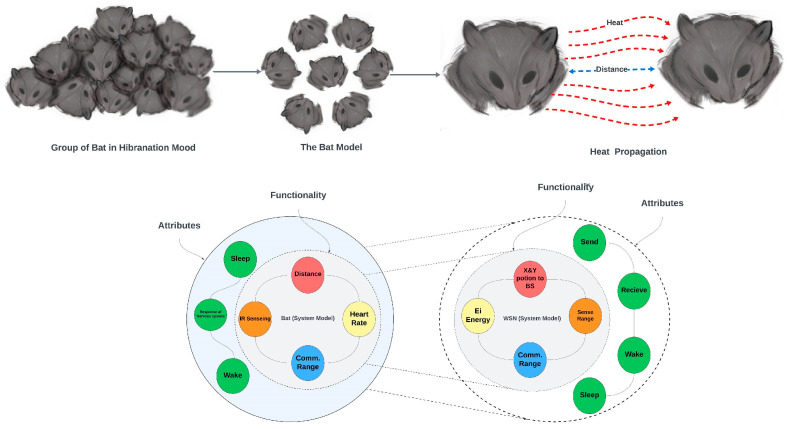
Integration of the bat model into the WSN model.

The standard bat algorithm is a heuristic search-based algorithm that emerged from the swarm search optimization technique, in which hunting for prey through the echolocation behaviors of bats is simulated. In this work, a new bat node scheduling algorithm was inspired by the behavior of bats when hibernating in the cold mountains to preserve energy [13,31,32]. Hibernating is the process of going into a deep sleep. Certain groups of bats hibernate by grouping themselves, reducing their temperature, slowing their heartbeat rates and breathing rates significantly, and expending little energy. When the bat group decides to wake up, the bat in the middle of the group accelerates its heartbeat, which in turn accelerates its blood circulation, causing heat to be produced. Finally, the heat is propagated from the closest bat to the rest of the bats in the group, where they start waking up gradually. Figure 1 maps the bat-inspired algorithm to the WSN model.

Nature-inspired algorithms (NIAs) have shown promising results in solving optimization problems [19]. In particular, the organization of bats and their energy-conservation strategy can serve as a model for WSNs. In Figure 1, the variables or parameters of the proposed bat model are mapped into the specification of the sensor nodes model. Therefore, in this work, the distance (X and Y positions to the BS) is interpreted as connectivity, and the heart rate ($E_{i}$ Energy) is referred to as lifetime. Hence, there are four main parameters introduced for the bat-based node scheduling algorithm, namely: $E_{i}$ Energy, X and Y positions to the BS, Sense Rang, and Comm Rang. The formulation of the problem is presented in the context of WSNs below.

### 3.1. Problem Formulation

In this study, we draw a parallel between the hibernation process of bats and the deployment of WSN nodes to formulate the problem. The bat population has the ability to form clusters during deep sleep, coupled with a variation in their heart rate. The heart rate of a bat is interpreted as the residual energy of a WSN node, as illustrated in Figure 1. In other words, when a bat is in a sleep state, the heart rate is reduced, and when a bat is in a waking-up state, the heart rate is increased. This variation in heart rate is interpreted as low and high residual energy in sensor nodes. Thereby, two factors are extrapolated: positions and energy. The higher energy level is required to wake the farthest bats, and the position can be mathematically determined by adjusting the energy levels. The novel approach proposed in this paper stems from adding convergence time and energy to the multicore space search problem, where an objective function is formulated using Equation (6) [33]. The proposed bat node scheduling algorithm generates solutions randomly to form the search space, where a loop search is used to find the optimal solution. The constructed search space is composed of several solutions, where the optimal solutions are listed.

The general structure of the bat algorithm is borrowed from the work in [16]. Accordingly, there are four dimensions of the solution space that the proposed bat node scheduling algorithm takes as inputs: a fixed frequency $f_{min}$, a velocity $V_{i}$, a position $Z_{i}$, and a heart rate $E_{i}$. The updated output values for energy, position, and heart rate with fitness function were calculated as described in Equations (4) and (5). Successively, Pareto optimization is used with the output values of the bat node scheduling algorithm to visualize and organize the solutions in a meaningful manner. Let the coordinates of a bat under consideration in the search space be ($Z_{i}$, $E_{i}$), where $Z_{i}$ is the position with respect to time, e.g., the position with reference to the base station, and $E_{i}$ is the energy of the bat with respect to time. The distance between nodes and the base station is also a contributing factor to the search space. We can write the following Equation (1) as:

(1) Z(t+1)=Z(t)+E(t+1)

(2) E(t+1)=E(t)+(Z(t)–k(t))

Now that the solution is obtained in the search space, we need to make sure these solutions are within a certain range/loudness (i.e., the loudness is related to the amplitude of the wave generated by bats. The factor C is used to bring the loudness within the specific range). Where k(t) is a proposed global solution, the energy is depleted with respect to time because the sensor battery is drained after each cycle. Unlike the standard bat algorithm, which uses echolocation to generate loudness, the modified hibernation bat node scheduling algorithm uses the heart rate to generate waves, so this heart rate has an amplitude equal to its loudness. The difference in $f_{max}$ − $f_{min}$ is considered one, where f is the maximum and minimum value for the bat population, i.e., the generated solutions.

(3) $$A=f_{min}+\left(f_{max}-f_{min}\right)\times C$$

(4) $$C=\left(A-f_{min}\right)/\left(f_{max}-f_{min}\right)$$

where  A is the amplitude of the heart rate of the bat under consideration and C is a gradient (i.e., steepness or the slope of a curve in search space) average position. Hence, the solution proposed in this paper will have both local and global solutions. So, the local solution is:

(5) Z(t+1)=Z(t)+b(t)×P(t)   

where P(t) is the population density of bats and b(t) can be a varying constant. As the bats are clustered by nature, they tend to be dense inside and sparse outside, as explained in Equation (5). The global solution can be obtained by adding the P(t) variable to the local solution as described. The population P(t) can be averaged out by the following relation, representing the global solution:

(6)  P(t+1)=C×P(t)

The global solution is obtained with respect to variation in time, in which the correlation time between each sensor node is linked with the current level of energy. Therefore, it triggers multiple solutions rather than a single solution.

The proposed algorithm processes the residual energy of bats/nodes as a function of ON/OFF values with respect to time. Since energy depletes as time naturally progresses in the WSN, the residual energy is a proportional attribute to time, and the ON/OFF values will be dynamically updated by the proposed algorithm. The premise of our work is based upon previously calculated data, which contain sleep/awake values and energy, communication requirements, and scheduling values. The core of the proposed algorithm is to optimize the objective function related to the product of energy, distance, and network lifetime. In our solution, we consider the objective function, which considers its optimization goal energy (related to the network and lifetime), distance (related to connectivity), and network lifetime (related to scheduling). Algorithm 1 describes the functionality of our proposed bat solution.
**Algorithm 1.** Bat node scheduling algorithmInput: Initial population, frequency, and velocity of batsOutput: Global number of iterations.1: Initialize the bat population n, the heart rate *r*(*i*), and the velocity *v*(*i*)2: While (t<max_iteration)
3: $\;f_{i}=f_{min}+\left(f_{max}-f_{min}\right)\times \beta$ as in Equation (4)4: $v_{i}\left(t+1\right)=v_{i}\left(t\right)+\left(x_{i}\left(t\right)-x^{*}\right)$
5: $x_{i}\;\left(t+1\right)=x_{i}\left(t\right)+v_{i}\left(t+1\right)$
6:                                                          If $\left(rand>r_{i}\right)$
7:                                                          x(new)=x(old)+ε×fi
8:                                                          End If9:                     If $(rand<E)\;\&\&\;(z_{i}<Zsol)\;$
10:                   E(t+1)=∝× E(t)
11:                   z(t+1)=z(t)+b(t)×P(t) using Equation (5)12:                   End If13: Find the solution Zsol for $E_{i}$ using Equation (6)14: Rank the bats in P [] 15. Pareto-optimal (P [])16:    For each solution $S_{1}$ in S:17:                 Initialize a Boolean flag dominated = False.18:             For each solution $S_{2}$ in S:19:                 If $S_{1}$ is dominated by $S_{2}$ (all objectives of *S*_2_ are better or equal to *S*_1_),20:         set dominated = True and break.21:                 End If22:             If dominated is False,23:        add *S*_1_ to *P* as it is a Pareto-optimal solution.24:            End If25:                 End For 26: Return P as the set of Pareto-optimal solutions.27:    End For 28: End While

The objective function presents the possible solutions in the search space, whereas the fitness function is a function that analyzes a candidate solution to a problem. Therefore, to better understand the Algorithm 1 mechanism, three bat population-based scenarios are presented to demonstrate the process scheduling solution (see Table 1) for the MOO problem at hand.

### 3.2. Use-Case Scenario of the Bat Node Scheduling Algorithm

In this use-case scenario, a step-by-step explanation for three bat populations is considered to demonstrate the process of obtaining the optimal solution.

- The first step is the random initialization of sensor nodes, e.g., three populations with the energy value as stated in Table 1.
- The second step defines the heart rate, i.e., energy for the three bat populations, where the number of bats is determined, while the population density is represented by the number of bats/total test area. For example, suppose there are 100 bats and the test area is 100 square meters, then the population density is 100/100 = 1 bat per square meter (coverage). Table 1 is the first iteration, with random values assigned to the variables. In this step, the initialization takes place.
- The third step generates new solutions by adjusting the heart rate and updating the energy level with locations to generate the new solutions.
- The fourth step checks a condition (if ((rand)>c)=true;) and tests if there is a lower threshold value for each variable: frequency > 0, velocity = 0, no. of hops < number of nodes, and energy level ≤ max energy of the battery used in the sensor nodes. From the above, we choose Bat2 amongst the three bat populations because it has the highest frequency population with a moderate energy level; therefore, this condition makes it suitable to have better connectivity and coverage.
- The fifth step is the selection of the best solutions based on the fitness function defined by Equation (5). The fitness function operates as follows:
  Fitness function: z(t+1)=z(t)+b(t)∗p(t), where *t* is time in seconds. Now, assuming t is equal to 1 *s* and *b* is a constant value set to 0.02, the fitness function is updated as follows:
  $$\begin{array}{ll} z\left(2\right) & =z\left(1\right)+b\left(1\right)\times p\left(1\right)\\ & =10+0.02\times 10\left(1+0.02\right)=10.2\;\;\;\;Bat1\;\left(1st\;population\right)\\ & =15\left(1+b\right)=15\left(1+0.02\right)=15.3\;\;\;\;Bat2\;\left(2nd\;population\right)\\ & =13\left(1+b\right)=13\left(1+0.02\right)=13.26\;Bat3\;\left(3rd\;population\right) \end{array}$$
- The sixth step shows that if the output of the fitness function has similar values for all bat populations, it will execute this step to recalculate from the beginning.
- The seventh step selects the bat population that has the lowest energy values to achieve this objective (the minimum operating energy of the sensor nodes). Our objective is to make the performance 100% efficient in utilization, which is our ideal optimal solution. For example, assuming a number of nodes is the minimum requirement to form a WSN, let us say the WSN has 70 nodes with minimum operating energy (condition). Assuming that 50 sensor nodes have met the condition, we must then tune up the energy for the 20 nodes to join.
- The eighth step updates the solution, where the energy level $E_{i}$ is increased and the $Z_{i}$ is decreased. This means that the more energy per node, the fewer nodes involved, and the less energy per node, the more sensor nodes involved. And that is the main constraint of the objective function, as it is energy-dependent.
- In the ninth step, after the Bat2 solution is accepted, the ranking of the best-to-worst solution is determined. $E_{i}$ is reset, and $Z_{i}$ is decreased. There is a solution generated after each iteration. This iteration is based on the bat population.
- In the tenth step, the Pareto algorithm filters the achieved solutions in the search space to find the optimal solution. This filtration process is based on proximity to the base station and energy. For example, as we know, out of all the input parameters, the following are the two important ones chosen logically from domain knowledge:
  o The proximity, e.g., how close the sensor nodes are to the base station (assigned weights of 60%), has a certain level of importance.
  o The energy levels of the sensor battery (assigned weights of 40%) are the two important conditions (1 and 2) that we consider in the Pareto algorithm. They are collectively defined as $S_{1}$ (a collection of favorable attributes). The conditions include:
    o Packet generation;
    o Type of sensor hardware characteristics;
    o Throughput.

The 3 conditions are then defined as $S_{2}$ collectively (a collection of not-so-favorable attributes); therefore, as we can see, the energy levels of Bat3 are higher if it is near the base station; otherwise, this will affect the sequence, followed by Bat2 and Bat1, hence the priority will be to choose this sequence. Finally, the best solution is communicated to the base station.

## 4. Experiments and Discussion

In this study, a simulation-based experiment is used to study and analyze the proposed bat node scheduling algorithm. In particular, the MATLAB simulator version R2018b-based event-driven programming simulator is used. A comparative analysis is used to investigate the performance of the newly proposed bat node scheduling algorithm and compare it against the HMM node scheduling algorithm and a normal non-scheduling cluster-head algorithm. The simulation is composed of rounds; in each round, there is an activity of sensing and reporting the sensed data back to the base station. The parameters used in this work include simulation parameters, coverage, and nodes, and the values are commonly used in the literature [7,13]. In this work, the same values are adopted for the simulator environment. There are three types of WSN communication ranges: short, long, and heterogenous. In this experiment, we have considered the use of the short-range ZigBee technology for the WSN, where its main features are the low data rates and low power consumption. We matched our communication range to be within 5 m. It is worth mentioning that the ZigBee communication range is very strong when the signal strength is within the 1 to 5 m range [34]. Furthermore, we adopted a realistic, commonly used energy model, as stated in [13]. Thus, in this experiment, the following assumptions and parameters are considered, as shown in Table 2.

### 4.1. Experiment Assumptions

**Figure 2:**
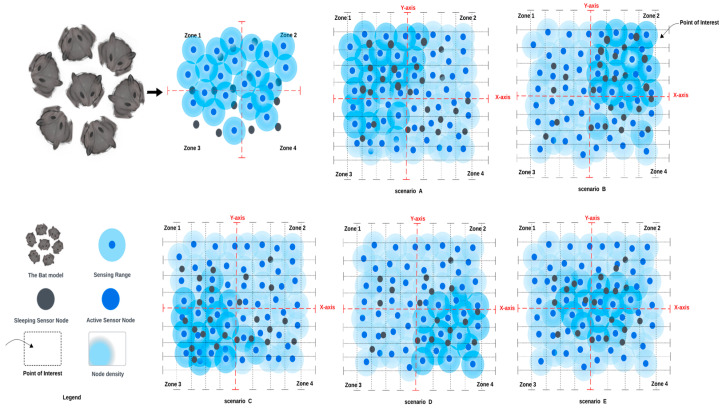
The spatial connected coverage scenarios (A, B, C, D, and E) over the area to be monitored.

In our work, we considered a uniform deployment with a random distribution over an area to be monitored. We have assumed that the area to be monitored is composed of multiple grids, where each grid represents the points of interest (POIs) [13]. In order to ensure that the event is detected and reported back to the base station, we assumed that the network coverage intensity was high and was based on the probability coverage model [19]. So, we defined a POI as the area to be covered by at least one sensor node in the WSN. Moreover, to ensure a good level of coverage, we defined our coverage metric to be greater than or equal to the area to be monitored. Consequently, we assumed that when connectivity between sensor nodes is established, all nodes are connected in the WSN. Thereby, there is always a maintained path from the base station to all nodes deployed over the area to be monitored. Furthermore, we defined the network lifetime as the most useful lifetime where sensor nodes maintain a level of connected coverage with the expected network operational lifetime over the area to be monitored. Figure 2 represents the possible spatial coverage scenario over the area to be monitored. In each scenario, the level of node density distributed randomly within a certain zone can be noted.

Accordingly, the bat node scheduling algorithm considers the solution in two steps:

1. local solution where the location Z and the energy E of each node are factored in to obtain the local solution during the run-time operation of the WSN.
  For example, let us assume that we have three nodes: A,B, and C, where the operation time t of node A is 1 s and the location of A is 3. Therefore, (1,3) is the local scheduling solution for A. Respectively, node B is (2, 6) and node C is (3, 9).
  3×1=3, 3×2=6, 3×3=9
2. Therefore, the global solution is obtained by adding the conversion time and energy to the multicore search space. Where the global solution is presented by (t, 3×t)
  when t =1; (1, 3×1)=(1×3)=3
  when t =2; (2, 3×2)=(2×6)=12
  when t =3; (3, 3×3)=(3×9)=27

In principle, the time variations, specifically the correlations in time between each sensor node, are incorporated, which are bound to the energy needed to predict the future operational time of each sensor sleep schedule.

**Figure 3:**
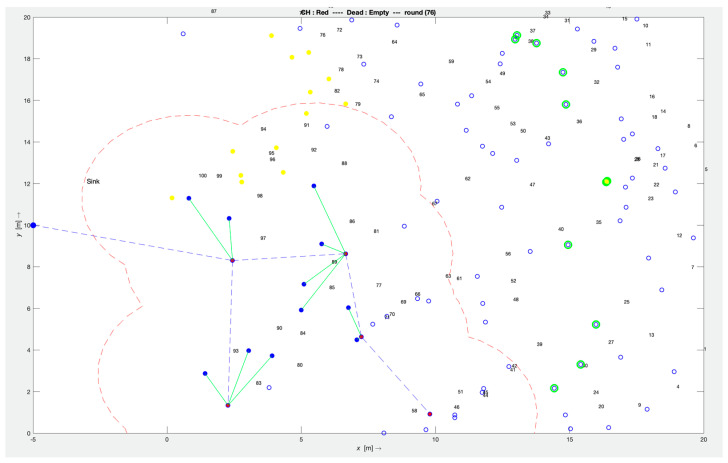
Simulation set-up for the bat node scheduling algorithm (Red—cluster head, Blue—child node, Yellow—sleeping node, Green—Overused node).

In the simulation, the experiment runs through 2000 rounds with a threshold to stop the simulation when the last number of nodes consumes all its energy. Figure 3 illustrates the experimentation set-up with the area to be monitored using 150 nodes. For statistical significance tests, each experiment simulation was individually tested 30 times. We implemented a one-way ANOVA to identify the *p*-value for statistical results from the bat node scheduling algorithm.

The output of the bat node scheduling algorithm is an optimal node schedule as presented in Table 3, where each node has an ON/OFF time instance.

For example, when time is arbitrarily assigned to each instant, such as Instant 1 = 1, Instant 2 = 2, Instant 3 = 4, and Instant 4 = 7, apply the following equations:

- Instant 2 − Instant 1 = 2 s − 1 s = 1 s;
- Instant 3 − Instant 2 = 4 s − 2 s = 2 s;
- Instant 4 − Instant 3 = 7 s − 4 s = 3 s.

The achieved results showed that the bat node scheduling algorithm outperformed the HMM node scheduling algorithm by 30% for the network lifetime, 40% for the network coverage, and 26.7% for the network connectivity. Furthermore, it is significantly better with respect to the parameters because p≤0.05. Table 4 represents the statistical analysis of the simulation experiments.

What follows represents the metrics we used as the base to compare our novel bat node scheduling algorithm, the HMM node scheduling, and the RCS algorithms. We denote the HMM as M, the RCS as O, and the bat node scheduling algorithm as Bat. For extra analysis assurance for our experiment, we simulated all the above algorithms with and without scheduling to observe both cases’ performance with respect to energy conservation.

### 4.2. All Used Energy

**Figure 4:**
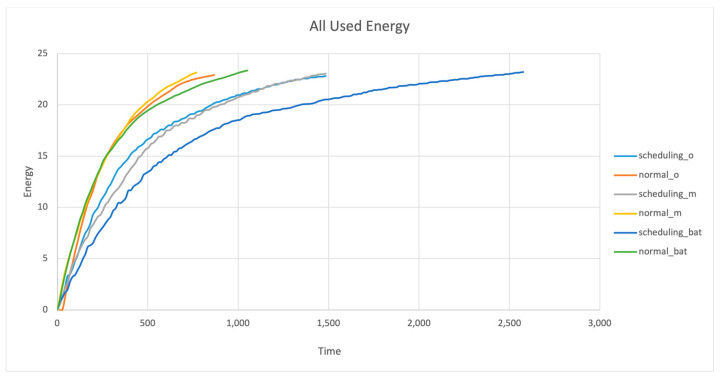
All used energy.

All used energy is a metric that investigates the network energy consumption throughout the simulation rounds. Figure 4 represents the usage of this metric, where the Y-axis is the nodes’ used energy and the X-axis is the round number. The energy efficiency of the bat node scheduling algorithm met the requirements and outperformed the HMM node scheduling and RCS algorithms. Due to its ability to produce a solution that has high performance in terms of coverage, connectivity, and network lifetime, the proposed bat node scheduling algorithm can be utilized to cover a wide range of safety-critical systems. For example, scenarios that can include forest fire detection systems, skyscraper fire detection systems, power plant nuclear systems, etc.

It can be noticed that the bat node scheduling algorithm reached an energy consumption of 24 joules at the 2555th round, while the HMM node scheduling algorithm scored it at the 1000th round. This shows how energy efficient the bat node scheduling algorithm is at conserving energy for more than double the time rounds achieved by the HMM node scheduling algorithm. As energy is one of the main critical parameters, using it cautiously is one of the prime requirements of the network. As described earlier, the bat node scheduling algorithm uses energy as one of the optimizing parameters due to the stable energy considerations in terms of distance, as depicted in Figure 3. However, in the HMM node scheduling and RCS algorithms, there was randomization of node allocations in the set-up phase to join WSN subsets; hence, their ON/OFF deployment will be based on that result. Additionally, there were extra ON nodes to fill the network’s coverage holes. When the sensor’s residual energy was not considered, the performance was compromised with respect to the RCS algorithm and HMM node scheduling algorithm. This result confirms that the residual energy information is useful in extending the time taken to the first depletion of node energy in the bat node scheduling algorithm.

### 4.3. Lifetime of Sensor Nodes

**Figure 5:**
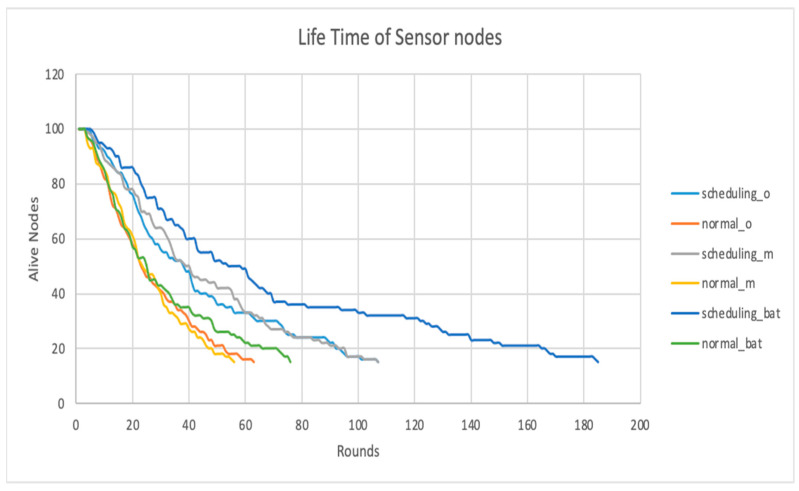
Lifetime of Sensor Nodes.

The lifetime of sensor nodes is a metric that investigates the number of alive nodes throughout the simulation rounds, where the Y-axis represents the number of nodes alive and the X-axis represents the number of rounds. The bat node scheduling algorithm improved noticeably in terms of nodes’ lifetime energy, as can be seen in Figure 5. Notably, in the bat node scheduling algorithm, the number of nodes that stay alive is 18 at 181 rounds; meanwhile, the HMM node scheduling algorithm recorded 18 nodes alive at 113 rounds. Consequently, the bat node scheduling algorithm outperformed the HMM node and the RCS algorithms by a noticeable margin.

The RCS algorithm uses a random coverage model utilizing an integrated method that provides statistical sensing coverage and guaranteed network connectivity. However, its lifetime was compromised in this experiment due to the randomization of allocating nodes in joining network subgroups. Furthermore, the RCS algorithm turns extra nodes ON to compensate for the coverage holes. This limitation is reflected in Figure 4, in which the light blue curve of the RCS algorithm recorded 18 live nodes at 101 rounds for the remainder of its lifetime.

Our proposed bat node scheduling algorithm solves these limitations by replacing the node allocation randomization with an objective function focusing on network lifetime, coverage, and connectivity during the network’s runtime. This objective function introduces pre-determined schedules for the nodes across their lifetime, enabling the nodes to be fully aware of their ON/OFF states without disturbances due to randomizations. This determinism in stochastic scheduling improves the network’s lifetime, coverage, and connectivity in the sense that these schedules identify the WSN’s optimal solutions, as illustrated in Figure 3. In support of this, Figure 4 clearly shows the optimal solutions, in which energy and distance were the considerations. It would be interesting to further analyze the Bat node scheduling algorithm using the Artificial Intelligence (AI) method [15]. Furthermore, when the bat node scheduling algorithm was implemented, coupled with the Pareto sorting algorithm, it provided those solutions in the space that were noticed to be stable. Our proposed approach here allowed us to sustain the process of dissemination of messages over the network for a longer time, e.g., sustain QoS levels of service availability and reliability.

### 4.4. Efficiency

**Figure 6:**
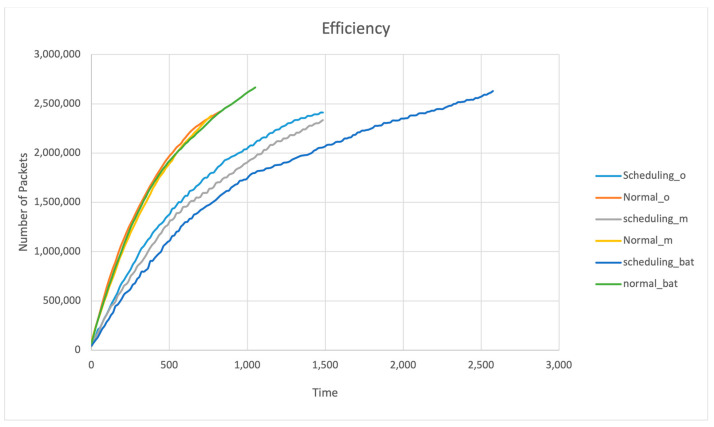
Efficiency.

During the lifetime of the network, there are a number of packets sent and received, and here we compare the throughput of all the mentioned algorithms with our proposed bat node scheduling algorithm. The correlation measured here is that the longer the network lifetime, the higher the number of packets sent and the receiving rate, hence a higher efficiency. Figure 6 represents the efficiency, where the Y-axis is the number of packets sent and received and the X-axis is the number of rounds.

It is noticeable from Figure 5 that the bat node scheduling algorithm has recorded a higher efficiency than the other algorithms. For example, the bat recorded 2,500,000 packets of throughput at the longest lifetime of the recorded simulation at the 2555th round. Since the bandwidth of the network is defined as the number of packets generated in the network, we can clearly say that lower bandwidth will result in less congestion and less information, and more bandwidth will result in more congestion and more information. Hence, we can see from the graphs that the bat node scheduling algorithm provides trade-offs between congestion and information. Hence, the efficiency of the network will be better compared to others. It can be noticed that the bat node scheduling algorithm has recorded the lowest packet generated (less congestion) from the 1st to 1000th round. The more packets are generated, the more they will use the maximum bandwidth available, causing energy to be lost due to excess packet generation and hampering other functions like connectivity coverage and lifetime.

### 4.5. Connectivity

**Figure 7:**
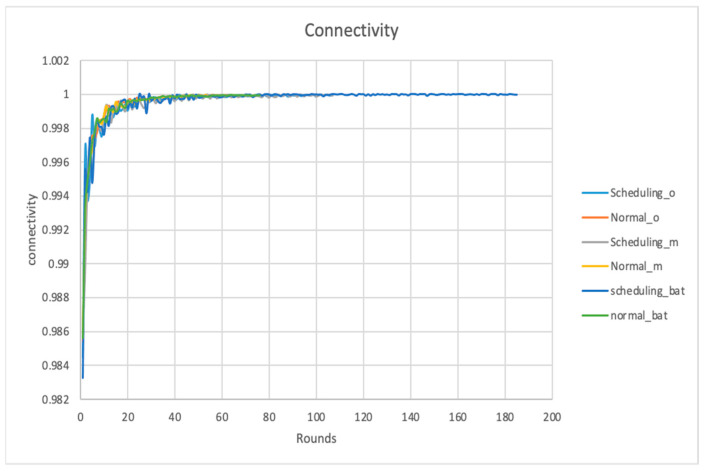
Connectivity.

In this metric, we are investigating the connectivity of the sensor nodes in the network. In Figure 7, the Y-axis is the performance of connectivity, which is measured by a number as a value, e.g., the fraction of nodes connected in the network, and the X-axis is the time, which is measured by the number of rounds. Here, 150 nodes in the network are considered; if the connectivity is 0.98, then the total connected nodes are calculated by 150×0.98=149.

As can be noticed from Figure 6, from the 140th round to the 158th round, the bat node scheduling algorithm recorded a connectivity value of 1, which means that the network is live and has better connectivity in comparison to the other algorithms. This observation shows the bat node scheduling algorithm has achieved the highest connectivity measurement with respect to the number of bats/nodes optimized energy E and the distance Z manifested in the prolonged network lifetime. The network lifetime was defined as the time slots passed until there was no set of active nodes that covered all points of interest or guaranteed connectivity between the sink and sensing nodes. As E and Z reached the stable point, as shown in Figure 6, the average lifetime also increased.

### 4.6. Coverage

**Figure 8:**
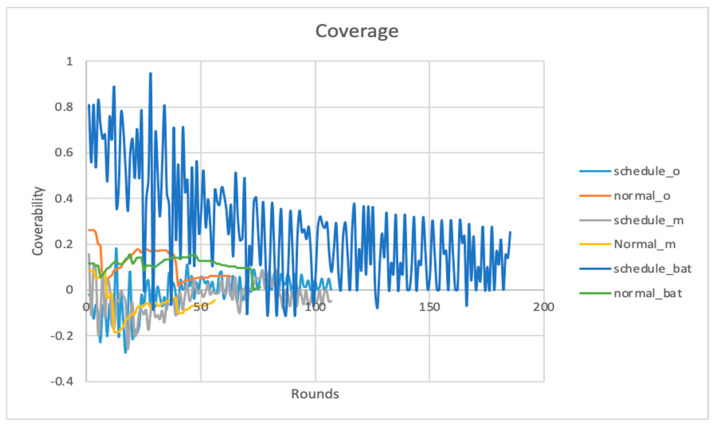
Coverage.

Coverage is a metric that measures the ratio of all node coverage to the area to be monitored. In Figure 8, the Y-axis is the ratio of the coverage to the total area to be monitored, and the X-axis is the number of rounds.

As can be noted, the bat node scheduling algorithm has improved coverage at the 180th round. Meanwhile, the HMM node scheduling recorded a coverage of 0 at round 100 because the nodes there had already lost their coverage since they prematurely depleted their energy reserves. In the case of coverage, we have the fraction of the area covered by the network. If the area to be monitored is 100$\;m^{2}$, a coverage of 0.8 would mean 0.8×100=80 $m^{2}$ of that area has been covered.

## 5. Conclusions

In order to improve the coverage, connectivity, and network lifetime of WSNs, many scheduling algorithms are possible. However, how good an algorithm is or if it is the best is not easy to answer. Bio-inspired computation opened the way for search space exploration; hence, our main contribution is determining whether a bat node scheduling algorithm can find a better solution than one proposed by an earlier HMM node algorithm. The novel, proposed bat node scheduling algorithm has improved network lifetime, which in turn has improved service availability for more dependable WSNs. The bio-inspired bat node scheduling algorithm prevents the languishing of the values within the local maximum and local minimum. Instead, it provides stable global maximum and minimum values that remain the same throughout the lifetime of the algorithm. In addition, from an algorithmic point of view, the bat and Pareto optimization algorithms worked in tandem to improve the performance of network parameters like connectivity, coverage, and network lifetime. Our results show significant improvements in all three dimensions, and the Pareto sorting guarantees us that the proposed bat node scheduling is the best for all possible fitness and objective functions and for a given search space. This leaves open the possibility for another algorithm to outperform our bat node scheduling algorithm. The network lifetime, coverage, and connectivity were improved, contributing to the dependability of WSNs. Assumptions/simulation parameters were used to make the work presented here more relevant to real-time WSN scenarios. Hence, our pursuit of the best scheduling algorithm for a WSN is not over yet. It would be equally interesting to implement this algorithm in mobile ad hoc network (MANET) or vehicular ad hoc network (VANET) environments. 

## Figures and Tables

**Table 1:** Initializing the bat population with random values.

Bat1	Bat2	Bat3	Variables	c Threshold Value
10%	15%	13%	Frequency f (percentage or population of nodes ON)	f>0
0	0	0	Velocity v (mobility status)	v=0
1	3	5	Position *N* (no. of hops)	*N* < number of nodes
25	26	30	Heart rate *E* (energy)	*E* ≤ 30 J

**Table 2:** Simulation parameters.

Parameter Name	Value
Length of the network (m)	*W* = 20
Width of the network (m)	*L* = 20
Initial energy of each node (joules)	*Ei* = 0.25
Packet size for cluster head per round (bits)	*CHpl* = 3000
Desired percentage of cluster heads	*p* = 5/100
Max number of simulated rounds	*num_rounds* = 2000
Average time in seconds taken in the setup phase	*Tsetup* = 4
Average time in seconds taken in the steady-state phase	*Tss* = 10
Energy for transmitting one bit	*Etrans* = 1.0000 × 10^−9^
Energy for receiving one bit	*Erec* = 1.0000 × 10^−9^
Data aggregation energy	*Eagg* = 1.0000 × 10^−9^
Energy of the free space model amplifier	*Efs* = 5.00 × 10^−8^
Distance from the sink to the sensor area	*Marg* = 5
Packet size for normal node per round (bits) for each type	*rates* = 80
Max range for wireless transmission for each node	*R* = 3.5

**Table 3:** Node scheduling with variant ON/OFF time instants.

Nodes ID	Instant 1	Instant 2	Instant 3	Instant 4
Node 1	OFF	ON	OFF	ON
Node 2	ON	OFF	ON	OFF
Node 3	OFF	OFF	ON	ON
Node 4	ON	ON	OFF	OFF

**Table 4:** Statistical analysis.

Metrics	Bat Node Scheduling Algorithm	HMM Node Scheduling Algorithm
Number of hubs	26	20
Connectivity	200	181
Coverage	0.98	0.97
Lifetime	196	183
ANOVA 1	$p=4.0669\times 10^{-175}$	$p=5.22\times 10^{-156}$

## Data Availability

Data are contained within the article.
